# Leaf antioxidant activity in Colombian elite *Hevea brasiliensis* genotypes as a breeding strategy for water deficit tolerance under Amazonia conditions

**DOI:** 10.1371/journal.pone.0306083

**Published:** 2024-09-12

**Authors:** Lised Guaca-Cruz, Armando Sterling, Andrés Clavijo, Juan Carlos Suárez-Salazar

**Affiliations:** 1 Doctorado en Ciencias Naturales y Desarrollo Sostenible, Facultad de Ciencias Agropecuarias, Universidad de la Amazonía, Florencia, Caquetá, Colombia; 2 Laboratorio de Fitopatología, Instituto Amazónico de Investigaciones Científicas Sinchi–Facultad de Ciencias Básicas—Universidad de la Amazonía, Florencia, Colombia; 3 Programa de Ingeniería Agroecológica, Facultad de Ingeniería, Universidad de la Amazonia, Florencia, Colombia; 4 Centro de Investigaciones Amazónicas CIMAZ Macagual César Augusto Estrada González, Grupo de Investigaciones Agroecosistemas y Conservación en Bosques Amazónicos-GAIA, Florencia, Colombia; Central Research Institute for Dryland Agriculture, INDIA

## Abstract

This study evaluated the foliar antioxidant activity in nine *Hevea brasiliensis* genotypes from the ECC-1 (Élite Caquetá Colombia) selection and IAN 873 cultivar (control) in trees in the growth stage in two large-scale clonal trials in response to different climatic (semi-humid warm and humid warm sites) and seasonal (dry and rainy periods) conditions in the Colombian Amazon. The results indicated that Reactive Oxygen Species (ROS) production increased under conditions of lower water availability (dry period), leading to lipid peroxidation, high defense of photosynthetic pigments, and development of better osmotic adjustment capacity in the ECC 64, IAN 873, ECC 90, and ECC 35 genotypes due to high concentrations of carotenoids (0.40 mg g^-1^), reducing sugars (65.83 μg mg^-1^), and malondialdehyde (MDA) (2.44 nmol ml^-1^). In contrast, during the rainy period, a post-stress action was observed due to high contents of proline and total sugars (39.43 μg g^-1^ and 173.03 μg g^-1^, respectively). At the site level, with high Photosynthetically Active Radiation (PAR) values (1143 moles photons m^-2^ s^-1^), temperature (32.11°C), and lower precipitation (135 mm), higher antioxidant activity (chlorophylls a, b and total, carotenoids, and proline) was recorded at the humid warm site, demonstrating that the ECC 90, ECC 64, and ECC 66 genotypes are tolerant to water deficit compared to IAN 873. The ECC 64 genotype, independent of seasonal changes and site conditions, presented the highest contents in Chl a, total Chl, reducing sugars, total sugars, and MDA, showing a tendency to adapt to fluctuating conditions. This study showed that water fluctuations do not cause the same metabolic responses, these vary within the same species, depending on their developmental stage and the climatic and seasonal variations characteristic of the Colombian Amazon.

## Introduction

The rubber tree [*Hevea brasiliensis* (Willd. Ex Adr. de Juss.) Muell.-Arg], native to the Amazon rainforest of Brazil, Bolivia, Peru, and Colombia, is cultivated in other tropical regions of South America, Asia, and Africa [1]. It is among the main economically, commercially, and socially significant tree crops worldwide [2]. In 2022, 14.6 million tons of natural rubber were produced [3], with its most important use being industrial for the manufacturing of tires [4] followed by multiple finished products [5, 6]. Colombia contributes 0.03% of the world’s production [7], coming from the departments of Meta, Caquetá, Santander, and Antioquia [8].

For the cultivation of rubber crops, some optimal environmental conditions are required, mainly temperatures ranging from 22°C to 30°C, an altitude of 1,300 meters above sea level [9], high relative humidity of 80%, with radiation levels from 1747 to 2600 hours per year, and six hours per day in all months [10], are among others that positively affect production. However, this can be altered by water availability, in which higher availability of moisture during wet months and lower availability during dry months [11] or very sunny periods with high humidity during the rainy season, imposing water deficit conditions [12].

Rubber plantations consume high levels of water [13, 14]. The main source water rainfall, during the dry season, water supplies become unstable [15]. During the rainy season, water supply may not be sufficient due to rubber trees’ dependence on shallow soil water [16] and the high transpiration rates due to the increased number of leaves during this period [17].

Water deficiency alters morphological and biochemical characteristics [18] and induces the biosynthesis of reactive oxygen species (ROS) that cause oxidative damage [19] to biological membranes and macromolecules (DNA, proteins, lipids, and photosynthetic pigments) [20]. In chlorophylls a and b, indirect photooxidation occurs under stress conditions due to damage to the photosynthetic apparatus [21] and to the activities of key enzymes in the Calvin cycle [22]. To neutralize the damage, plants increase their antioxidant defense [23], which can involve enzymatic and non-enzymatic mechanisms [24–26].

The activation of the natural defense system in the form of antioxidant enzymes and accumulation of osmolytes (amino acids, sugars, pigments) plays an important role in regulating photosynthesis. Carotenoids, for example, minimize the production of singlet oxygen [27] by dissipating excess light energy as heat [28], generating breakdown products as signalers [29]. Meanwhile, catalase (CAT) degrades hydrogen peroxide (H_₂_O_₂_) generated during mitochondrial electron transport into H_₂_O and O_₂_, while proline can quench both singlet oxygen (^1^O_₂_) and hydroxyl radicals (OH•) [30]. This is associated with the plant’s ability to cope with various types of stress [31, 32]. Similarly, carbohydrates are linked to adaptation and development processes under stress conditions [33], with their accumulation demonstrating greater tolerance to dehydration [34].

The metabolic/biochemical responses of plants have been used as important criteria to understand the extent of the interaction between plants and the atmosphere under various microclimatic conditions [35]. Seasonal variations in biochemical parameters can help to understand the adaptive capacity of rubber tree genotypes to changes in atmospheric temperature, rain, humidity, among others. Therefore, the objective of the study was to analyze the seasonal changes in the foliar antioxidant activity of nine elite Colombian *H*. *brasiliensis* trees and IAN 873 in the growth stage in large-scale clonal trials, to be used a strategy to improve drought tolerance under different environmental conditions of the Colombian Amazon.

## Materials and methods

### Study area

The study was conducted in two sites with different climatic characteristics, featuring undulating terrain and slopes not exceeding 25% in the department of Caquetá, in the northeastern Colombian Amazon region. The first site was El Paujil, in the rural settlement of Moravia (1°31’38.46’’ N and 75°17’32.59’’ W, at 282 meters above sea level), and the second site was San Vicente del Caguán, in the rural settlement of Buenos Aires (2°2’40.8’’ N and 74°55’11.7’’ W, at 346 meters above sea level). No permits were required for the research because it is an inter-institutional agreement between the Amazonian Institute of Scientific Research SINCHI and ASOHECA (Association of Reforesters and Rubber Growers of Caquetá).

### Climate

Caquetá has a humid tropical climate with an average annual temperature of 25.5°C, an average relative humidity of 84.3%, and an annual precipitation of 3793 mm. It has a monomodal precipitation pattern with: a dry period stretched November to February and a rainy period from March to June. The remaining months correspond to a transition between the rainy and dry periods [36].

El Paujil has a humid warm climate, with an average temperature of 25.8°C, an average relative humidity of 81.2%, annual precipitation of 3490 mm, and a Lang index of 135. In contrast, San Vicente del Caguán has a semi-humid warm climate, with an average temperature of 25.4°C, an average relative humidity of 79%, annual precipitation of 2503 mm, and a Lang index of 98.6.

### Soils

The soils of Caquetá exhibit a high degree of chemical, physical, and biological degradation with high compaction, acidity, aluminum saturation, aluminum ferric oxides, low fertility levels, cation exchange capacity, exchangeable bases, low phosphorus availability, and clayey texture [37].

The soils of El Paujil have an extremely acidic pH (4.91 units of pH), an electrical conductivity of 0.07 dS m^2^, a cation exchange capacity of 11.16 meq 100 g^2^, an organic matter content of 2.23%, a clayey texture (52.75% clay, 21.13% sand, and 26.13% silt), a total nitrogen content of 0.11% and aluminum saturation of 83.7% [38].

In San Vicente del Caguán, the soils have a very acidic pH (5.05 units of pH), high aluminum saturation (77.96%) with low contents of organic carbon (0.71%), organic matter (1.21%), available phosphorus (0.84 mg/kg), and total nitrogen (0.06%) with exchangeable base saturation of 4.99%; 2.23%; 1.18%; and 17.51% for Mg, Na, K, and Ca respectively. The concentrations of micronutrients are 161.59 mg kg ^−1^ for Fe; 4.27 mg kg ^−1^ for Mn; 1.71 mg kg ^−1^ for Cu; and 0.54 mg kg ^−1^ for Zn, with a loamy clay-sandy textural class (23.04% clay, 52.15% sand, and 24.81% silt) [39].

### Plant material

The plant material consisted of young 3-year-old trees (pre-tapping phase) from nine elite Colombian *H*. *brasiliensis* from the ECC-1 series (Elite Caquetá Colombia, ECC 25, ECC 29, ECC 35, ECC 60, ECC 64, ECC 66, ECC 73, ECC 83, and ECC 90), and the widely cultivated cultivar IAN 873, which is extensively grown in countries like Colombia [40]. The elite genotypes were obtained from a breeding program initiated by the Amazonian Institute of Scientific Research SINCHI, using a local germplasm collection (99 elite open-pollinated trees) in Caquetá. The elite genotypes were initially studied using morpho-agronomic and molecular descriptors in small-scale clonal trials [41], and subsequently evaluated in large-scale clonal trials [42]. The elite genotypes of the ECC-1 series have shown good performance in growth, early yields, disease resistance (40), and high photosynthetic performance [38].

### Experimental design

At each site, a Large-Scale Clonal Trial (LSCT) was established on 5.04 ha, The genotypes (9 elite genotypes and one control commercial genotype, IAN 873 control) were planted in a randomized complete block design with a control four replications (plots). Each plot covered an area of 1260 m^2^ and consisted of 60 trees per genotype arranged in three rows of 20 plants, with a planting frame of 7.0 m x 3.0 m, providing a density of 476 trees ha^-1^. Weed control was conducted every three months, and fertilizers were applied every six months: one application of 150 g plant^-1^ of N (15%), P_2_O_5_ (15%), K_2_O (15%), CaO (2.2%), SSO_4_ (1.7%), and another of 75 g plant^-1^ of N (8%), P_2_O_5_ (5%), CaO (18%), MgO (6%), S (1.6%), B (1%), Cu (0.14%), Mo (0.005%), Zn (2.5%), along with 1000 g of organic matter per plant [43].

### Biochemical responses

At each period, a sampling of three healthy leaves from the middle third of the canopy of ten tree per plot (genotype), block, and LSCT (Site) similar to that reported by Sterling et al. [38] between 9:00 and 12:00 h. This is the time range with the highest photosynthetic performance and maximum water use efficiency (36). These leaves were immediately frozen in liquid nitrogen and stored at -80°C until processing. Various tests were performed to determine the content of chlorophylls (Chls) and carotenoids (Car), reducing and total sugars, proline, malondialdehyde (MDA) content, catalase, and soluble protein.

Chlorophyll (Chls) and carotenoid (Car) contents were extracted in 80% (v/v) acetone and quantified spectrophotometrically according to Lichtenthaler [44]. Reducing sugar content was determined by the Nelson [45] -Somogyi [46] method for Cu^^2^⁺^ reduction and cuprous oxide Cu_₂_O production, which forms a complex quantified at 660 nm. For this, the macerated plant tissue was mixed with 50 mM C_₂_H_₃_NaO_₂_ buffer, pH 5.0, with constant agitation for 1 hour, then centrifuged at 6000 rpm for 30 min at 12°C for extraction. In a water bath at 90°C for 60 min, 50 mM C_2_H_3_NaO_2_ buffer pH 5.0, leaf extract, Somogyi I reagent (C_₄_H_₄_KNaO_₆_ *4H_₂_O; Na_₂_CO_₃_; Na_₂_SO_₄_) and somogyi II (CuSO_₄_*5H_₂_O; Na_₂_SO_₄_) were mixed, cooled to room temperature, then Nelson’s solution ((NH₄)₆Mo₇O₂₄ * 4 H₂O; H₂SO₄)) with distilled water was added for centrifugation at 14000 rpm for 60 min at 12°C, and absorbance was read at 660 nm against the blank [47]. Total sugar was measured according to the method of aldose and ketose formation in a strongly acidic medium [48]. Extraction was performed with distilled water with horizontal agitation for 60 min to separate the supernatant by centrifugation at 6000 rpm for 30 min at 12°C. Then, a solution was formed with distilled water, extract, 80% C₆H₆OH, concentrated H₂SO₄, exothermic reaction was cooled to room temperature, and absorbance readings were taken at 490 nm.

For the determination of proline content, the tissue was homogenized with 3% (w/v) sulfosalicylic acid, agitated horizontally for 60 min, and centrifuged at 6000 rpm for 30 min at 10°C. One ml of the supernatant was mixed with equal parts of acidic ninhydrin and glacial acetic acid. The mixture was agitated for 20 s, then heated in a boiling water bath for 60 min. After cooling, toluene was added, agitated for 60 s, and left to separate the phases. The chromophore was removed and analyzed spectrophotometrically at 520 nm [49]. Assays were performed in triplicate, and proline concentration was determined using a standard curve.

Malondialdehyde (MDA) content was quantified by the thiobarbituric acid (TBA) method [50]. Leaves were homogenized with 5% trichloroacetic acid (TCA) (5 ml), centrifuged at 15,000 rpm for 10 min. TBA (0.5%), 20% TCA, and the supernatant were mixed in equal volumes and incubated for 30 min at 100°C. Samples were centrifuged again at 10,000 rpm for 5 min. Finally, absorbances were recorded at 532 and 600 nm. For catalase determination, an enzymatic extract (0.15 g fresh leaves) was obtained with 50 mM sodium phosphate buffer, pH 6.8, to quantify the remaining peroxide (i): 1 ml of 50 mM sodium phosphate buffer pH 7.6, 250 μL of enzymatic extract, 600 μL of 3% H₂O₂ (v/v) (1.235M) in a test tube. After five minutes, 1 ml of 98% H₂SO₄ was added. In another container, 10 ml of distilled water and 1 ml of concentrated H₂SO₄ were added and heated to 75°C. The two solutions were combined and titrated with 10 mM potassium permanganate (KMnO₄) standardized to quantify available peroxide (ii): The same procedure was followed (remaining peroxide), considering that before adding the extract, 1 ml of 98% H₂SO₄ was added to inactivate the enzyme. Lastly, soluble protein content was quantified using bovine serum albumin (BSA) as a standard [51].

### Data analysis

The data for the different biochemical variables were analyzed using linear mixed models for longitudinal data. Site (humid warm and semi-humid warm), period (dry and rainy), genotype (nine elites and IAN 873), and their interactions were included as fixed effects, while blocks nested within sites and plots associated with genotypes within blocks were included as random effects. Assumptions of normality and homogeneity of variances were validated through exploratory residual analysis, and residual correlation was considered to analyze repeated measurements over time. Mean separation was performed using Fisher’s LSD test with a significance level of 5%. Principal Component Analysis (PCA) was used to study the relationships between the fixed effects and the different biochemical variables, and hierarchical cluster analysis based on Euclidean distance and Ward’s method was conducted to group the genotypes according to the biochemical variables. In addition, a chord diagram of Pearson correlation coefficients between biochemical variables of 10 rubber tree (*H*. *brasiliensis*) genotypes was created. The Linear Mixed-Effects (LME) models were fitted using the lme function from the nlme package [52] in R v. 4.0.3 [53], with the interface in InfoStat v.2020 [54]. PCA, hierarchical clustering and chord diagram were performed using Ade4 [55] and Factoextra [56], FactoMineR [57] and Circlize [58] packages from R v. 4.0.3, respectively.

## Results

**Fig 1:**
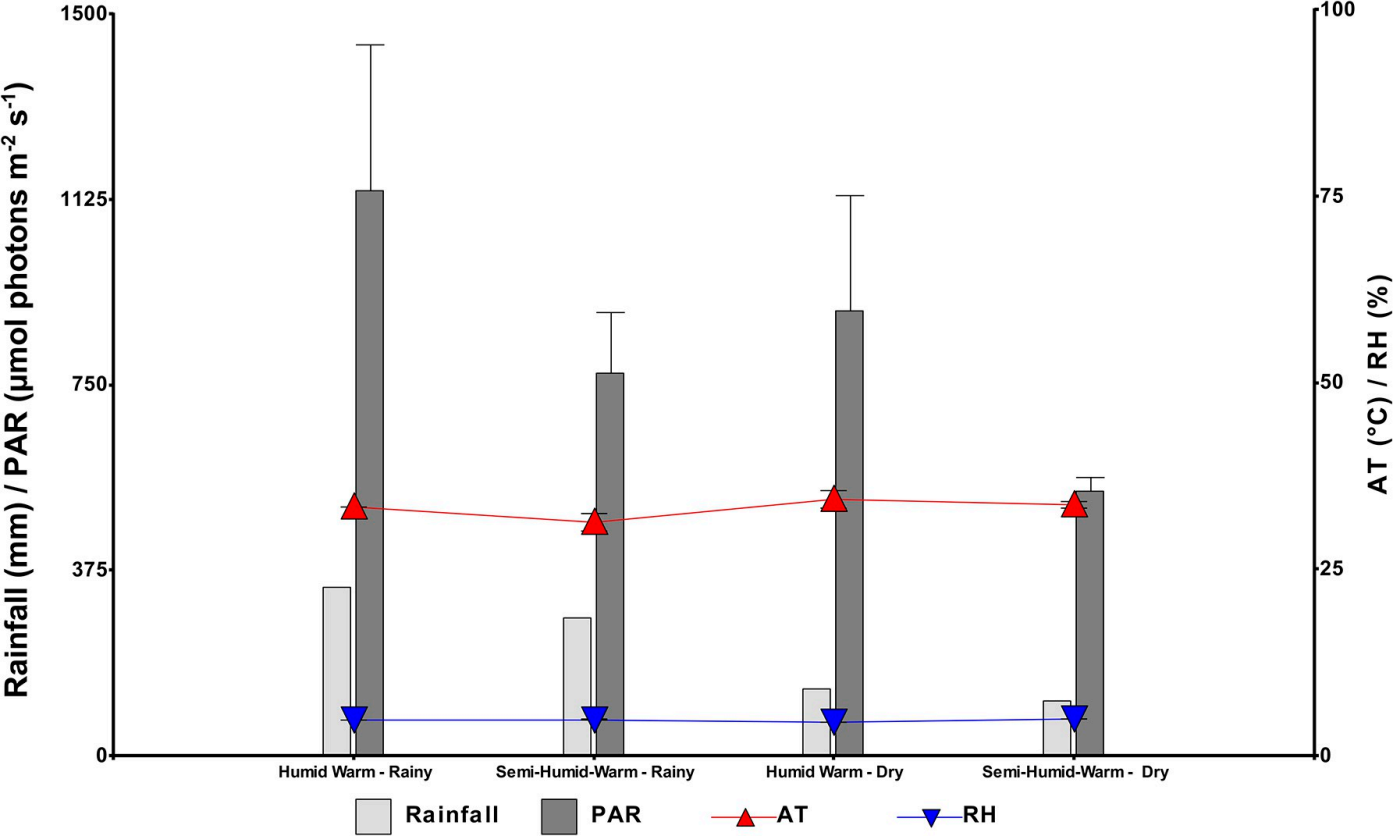
Monthly climatic data during the foliar sampling hours (9:00 and 12:00) showing climatic variations: Humid warm (El Paujil) and semi-humid warm (San Vicente del Caguán) in Caquetá with seasonal changes: Dry (February 2019) and rainy (May 2019). Photosynthetically active radiation (PAR), relative humidity (RH), air temperature (AT), and precipitation. Values represent the mean and error bars indicate the standard error (n = 95).

Precipitation and Photosynthetically Active Radiation (PAR) varied with the seasonal changes. During the dry season, the lowest precipitation was 135 mm recorded for El Paujil (humid warm) and 111 mm for San Vicente del Caguán (semi-humid warm) (Fig 1). The humid warm site exhibited the highest PAR values, with 1143 moles photons m^-2^ s^-1^ during the dry season and 899 moles photons m^-2^ s^-1^ was observed during the rainy season. In the semi-humid site, the highest relative humidity values were recorded for both periods, with 75% during the rainy season and 74% during the dry season. Air temperature showed similar behavior during the sampling months.

In the higher-order interaction, significant differences were observed for Chl a, protein, and carotenoids, the latter also showed a highly significant effect in the period, genotype, and all two-factor interactions. In contrast, the catalase enzyme was only influenced by the interaction of season and genotype (Table 1). Lipid peroxidation (MDA) showed highly significant variation for season, location, genotype, and the interaction between season and genotype. Overall, the effect of seasonal changes in climatic conditions on antioxidant activity was significant while site had a significant effect on chlorophyll content (*p* < 0.05).

**Table 1:** Analysis of variance of the fixed effects on the biochemical variables at the leaf level in nine genotypes of *Hevea brasiliensis* from the ECC-1 selection and the IAN 873 cultivar (control) across two seasonal periods and two sites with different climates in Caquetá (northwestern Colombian Amazon). Season (S), Location (L), genotype (G), and their interactions in chlorophyll a, b (Chl a; Chl b) (mg g^-1^), carotenoids (Car) (mg g^-1^), total chlorophyll (Chl total) (mg g^-1^), proline (μg g^-1^), catalase activity (CAT) (Ucat/mg protein), reducing sugars (μg mg^-1^), total sugars (μg g^-1^), protein (mg g^-1^), and malondialdehyde (MDA) (nmol ml^-1^).

Variables	*F* Based *p* Values						
	S	L	G	S X L	S X G	L X G	S X L X G
Chl a	0.6665	0.0005***	0.2351	<0.0001***	0.0007***	0.2811	0.0459*
Chl b	0.1887	0.0076**	0.1533	<0.0001***	0.0252*	0.7185	0.3665
Car	0.0026**	0.5931	0.0065**	0.0038**	0.0107**	0.0042**	0.0049**
Chl total	0.5295	0.0009**	0.0858	<0.0001***	0.0182*	0.5607	0.4293
Proline	<0.0001***	0.0149*	0.0434*	<0.0001***	0.2489	0.4468	0.2508
CAT	0.0971	0.2930	0.2431	0.0730	0.0379*	0.8011	0.9452
Reducing sugar	0.0021**	0.3359	0.7312	0.5024	0.8426	0.0383*	0.1605
Total sugar	0.0074**	0.6185	<0.0001***	0.0042**	0.8547	0.2069	0.2881
Protein	0.9583	0.3487	0.0071**	0.2777	0.0069**	0.0135*	0.0396**
MDA	<0.0001***	0.0009**	0.0008**	<0.0001***	0.8105	0.4200	0.1381

* Indicates significant differences * *P* = 0.05

** *P* = 0.01 and

*** *P*<0.001

The average content of carotenoids, reducing sugars, and lipid peroxidation (MDA) was high in the dry season, in contrast, proline and total sugars were significantly higher in the rainy season (Table 2). The results shown that the average content of photosynthetic pigments (Chl a and b, total), proline, total sugar and MDA were significantly higher in the warm humid location (El Paujil) whereas the average content of reducing sugar and protein were relatively higher in the semi humid location (San Vicente del Caguán). Among the genotypes, ECC 64 had the highest average of Chl a (1.01 mg g^-1^), total Chl (1.47 mg g^-1^), reducing sugars (64.91 μg mg^-1^), total sugars (206 μg g^-1^), and MDA (2.18 nmol ml^-1^) (Table 2).

**Table 2:** Mean values for chlorophyll a, b (Chl a; Chl b) (mg g^-1^), carotenoids (Car) (mg g^-1^), total chlorophyll (total Chl) (mg g^-1^), proline (μg g-^1^), catalase (CAT) (Ucat/mg protein), reducing sugars (μg mg^-1^), total sugars (μg g^-1^), protein (mg g^-1^), and malondialdehyde (MDA) (nmol ml^-1^) for each fixed effect studied.

Effect	Levels	Variables									
		Chl a	Chl b	Car	Chl total	Proline	CAT	Reducing sugar	Total sugar	Protein	MDA
Season	Rainy	0.98^a^	0.40^a^	0.36^b^	1.38^a^	39.43^a^	2.45^a^	52.56^b^	173.03^a^	3.91^a^	1.55^b^
	Dry	0.97^a^	0.43^a^	0.38^a^	1.40^a^	17.69^b^	3.31^a^	62.64^a^	144.20^b^	3.93^a^	2.06^a^
Location	Semi-humid warm	0.90^b^	0.34^b^	0.37^a^	1.24^b^	15.80^b^	2.59^a^	59.77^a^	150.37^a^	4.03^a^	1.38^b^
	Humid warm	1.04^a^	0.49^a^	0.37^a^	1.54^a^	41.32^a^	3.18^a^	55.43^a^	166.86^a^	3.81^a^	2.23^a^
Genotype	ECC 25	0.97^a-b^	0.41^a-b^	0.40^a^	1.38^a^	20.93^a^	3.30^a-b^	58.05^a^	162.63^c^	3.37^b^	1.63^b^
	ECC 29	0.98^a^	0.46^a^	0.38^a^	1.44^a^	37.47^a^	1.55^b^	51.15^a^	122.67^d-e^	4.64^a^	1.58^b^
	ECC 35	0.98^a^	0.40^a-b^	0.37^a^	1.38^a^	30.48^a^	2.47^a-b^	62.43^a^	199.40^a-b^	3.99^a-b^	1.79^b^
	ECC 60	0.87^b^	0.32^b^	0.32^b^	1.19^b^	28.12^a^	2.83^a-b^	58.63^a^	150.70^c-d^	4.03^a-b^	1.82^b^
	ECC 64	1.01^a^	0.46^a^	0.39^a^	1.47^a^	16.93^a^	2.78^a-b^	64.91^a^	206.00^a^	4.09^a-b^	2.18^a^
	ECC 66	0.96^a-b^	0.40^a-b^	0.36^a^	1.36^a^	33.47^a^	2.66^a-b^	59.18^a^	169.38^b-c^	3.31^b^	1.77^b^
	ECC 73	1.00^a^	0.41^a-b^	0.36^a^	1.41^a^	35.23^a^	2.25^a-b^	54.72^a^	113.68^e^	4.49^a^	1.67^b^
	ECC 83	0.96^a-b^	0.44^a^	0.36^a^	1.40^a^	23.32^a^	2.48^a-b^	55.52^a^	158.27^c-d^	3.92^a-b^	1.68^b^
	ECC 90	0.99^a^	0.46^a^	0.39^a^	1.45^a^	25.82^a^	3.95^a^	50.97^a^	165.68^a-b^	3.24^b^	2.16^a^
	IAN 873	1.00^a^	0.42^a^	0.37^a^	1.41^a^	33.83^a^	4.56^a^	60.46^a^	137.77^c-d-e^	4.11^a-b^	1.78^b^

**Fig 2:**
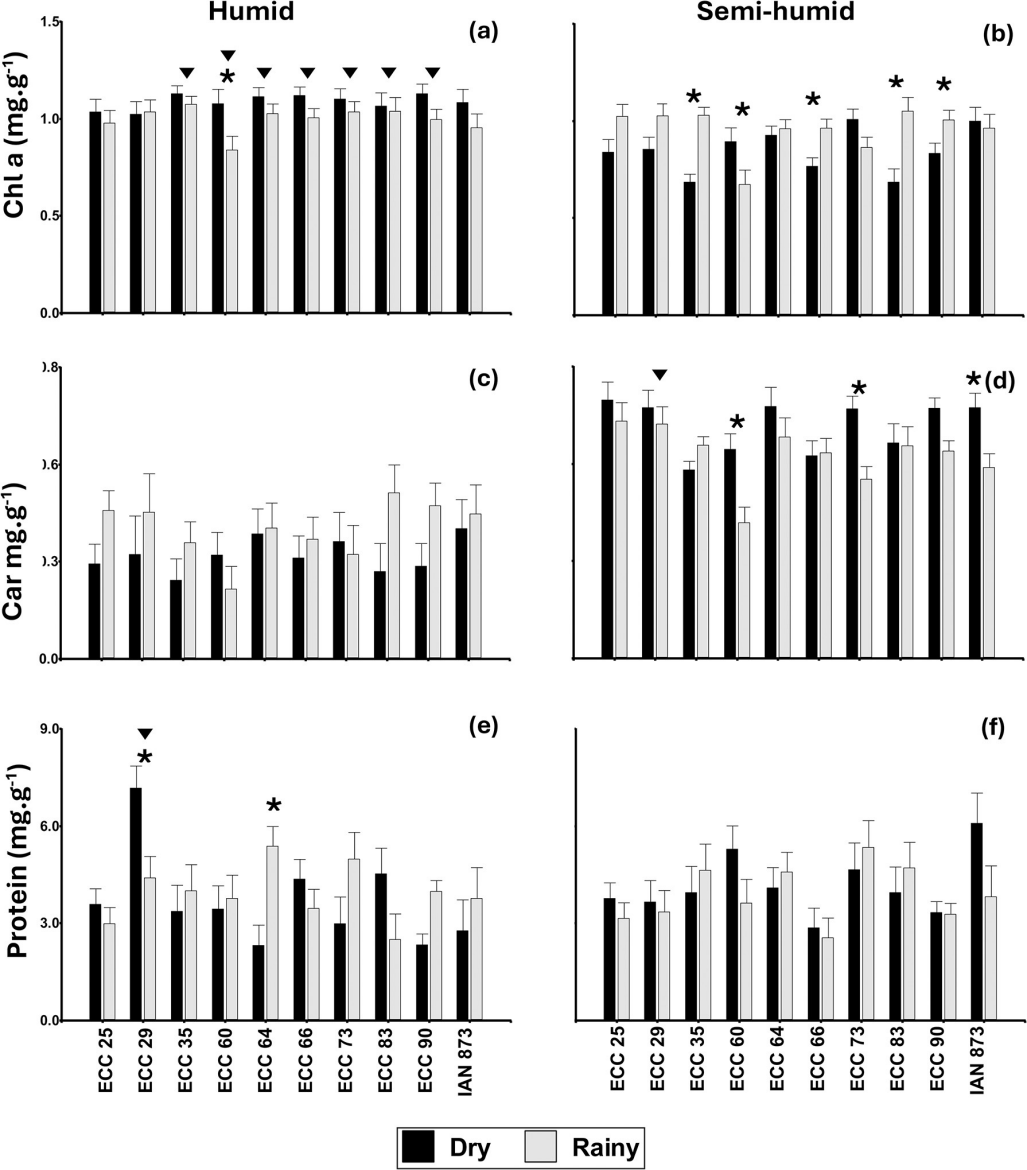
Changes in foliar biochemical parameters in 10 genotypes of rubber tree (*Hevea brasiliensis*) in two seasons and two sites with different climates in Caquetá (Colombia). (a) and (b), Chl a; (c) and (d), Car; (e) and (f), protein); (a), (c) and (e), humid warm site; (b), (d) and (f) semi-humid warm site. Means for warm humid and warm semi-humid locations are followed by an inverted triangle and, for dry and rainy season are followed by an asterisk (*) for each genotype, were significantly different according to Fisher’s LSD test, (*p < 0*.*05*). Values represent the mean ± SE of four replicates (n = 4).

The average value of Chl a for the humid warm location (El Paujil), it ranged from 0.84 mg.g^-1^ with genotype ECC 60 in the rainy season to 1.13 mg g^-1^ with genotype ECC 90 in the dry season (Fig 2A), while for the semi-humid warm location (San Vicente del Caguán), the range varied from 0.67 with genotype ECC 60 to 1.05 mg g^-1^ with genotype ECC 83 in the rainy season (Fig 2B). Genotype ECC 60 showed significant differences (*p* <0.05) at the season level in the humid warm site (Fig 2A).

The majority of elite genotypes exhibited variation in response to chlorophyll a in response to the site. Genotypes ECC 35, ECC 60, ECC 64, ECC 66, ECC 73, ECC 83, and ECC 90 recorded significant differences, while genotypes ECC 25, ECC 29, and control IAN 873 did not show differences (Fig 2A).

Carotenoids varied with seasonal and site dynamics depending on genotypes, with higher concentrations during the dry season, showing significant differences with genotypes ECC 60, ECC 73, and the IAN 873 (control) for the semi-humid warm location (Fig 2D). In contrast, for the humid warm location, carotenoid content remained relatively constant with minimal seasonal variation among genotypes (Fig 2C).

The protein content in leaf tissues was significantly higher (*p*<0.05) in the dry season and the humid warm site with genotype ECC 29 had the highest a mean value of 7.18 mg g^-1^ while genotype ECC 64 had higher protein content during the rainy season. (Fig 2E). The interaction of season, location, and genotype, did not show differences in protein content for the semi-humid warm location; however, the values ranged from 2.56 in genotype ECC 66 during rainy season to 6.08 mg g^-1^ in IAN 873 (control) in dry season (Fig 2F). Genotypes ECC 25, ECC 35, ECC 60, ECC 64, ECC 66, ECC 73, ECC 83, ECC 90 and IAN 873 (control) did not differ in mean protein content with climatic variation or location (Fig 2E and 2F).

**Fig 3:**
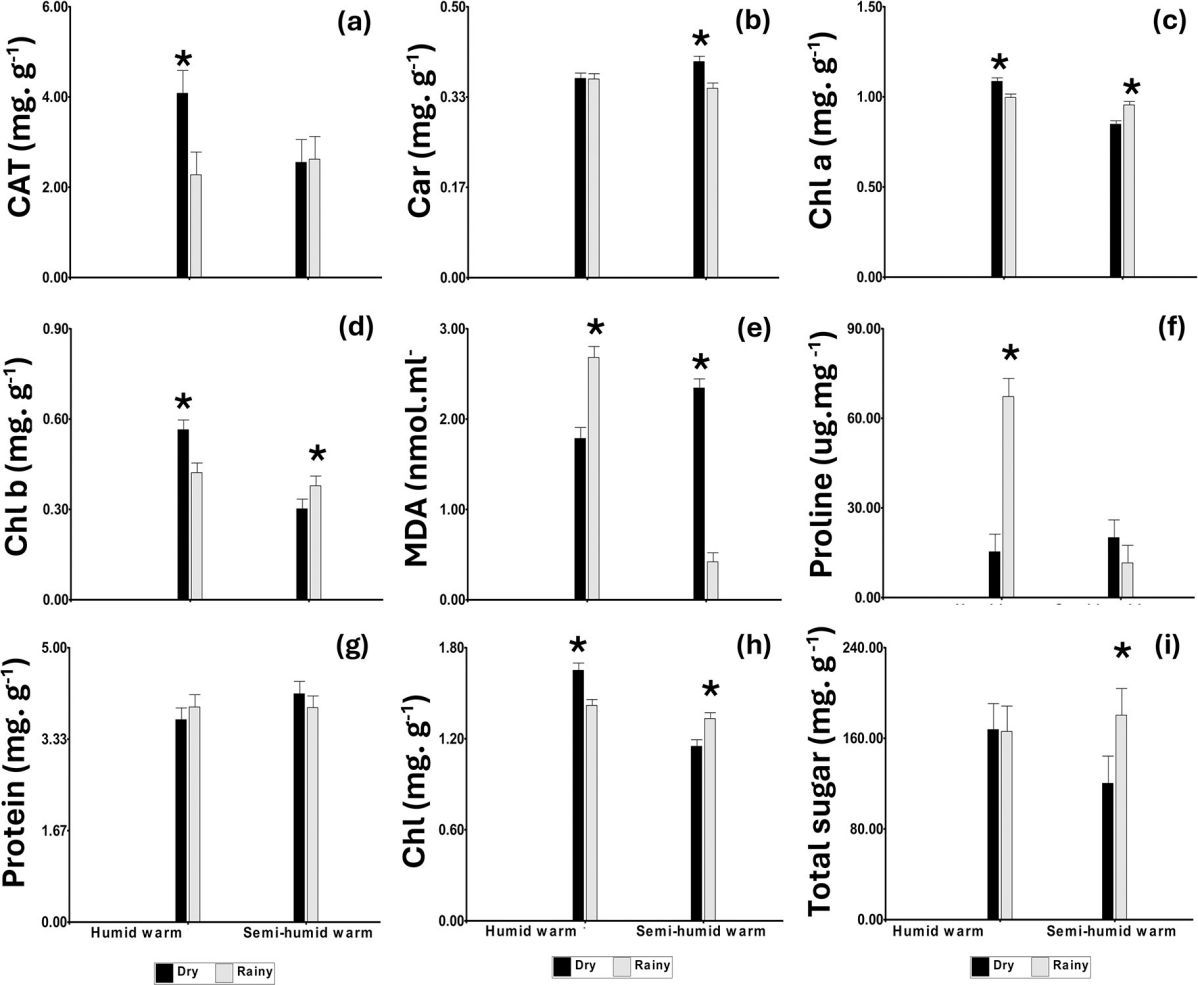
Variation of foliar biochemical parameters according to season and location conditions in Caquetá (Colombia). (a), CAT; (b), Car; (c), Chl a; (d), Chl b; (e) MDA; (f), proline; (g), Protein; (h), Chl total; (i), Total sugar. Means of dry and rainy seasons are followed by an asterisk (*) for each genotype, were significantly different according to Fisher’s LSD test, (p<0.05). Values represent the mean ± SE of four replicates (n = 4).

In the interaction between season and location, significant accumulations of CAT (4.08 mg g^-1^), Chl a (1.09 mg g^-1^), Chl b (0.56 mg g^-1^), and total Chl (1.65 mg g^-1^) were observed in the humid warm site during the dry season (Fig 3A, 3C and 3H). In contrast, during the rainy season, the content of MDA and proline were higher (Fig 3E and 3F). For the semi-humid warm site during the dry season, the foliar concentrations of Car (0.40 mg g^-1^), MDA (2.34 nmol ml^-1^), and total Chl (1.15 mg g^-1^) were significantly higher (Fig 3B, 3E and 3H) than those observed during the rainy season. However, in this season, the contents of Chl a, Chl b, and total sugars (180 mg g^-1^) were higher (Fig 3C, 3D and 3I).

**Fig 4:**
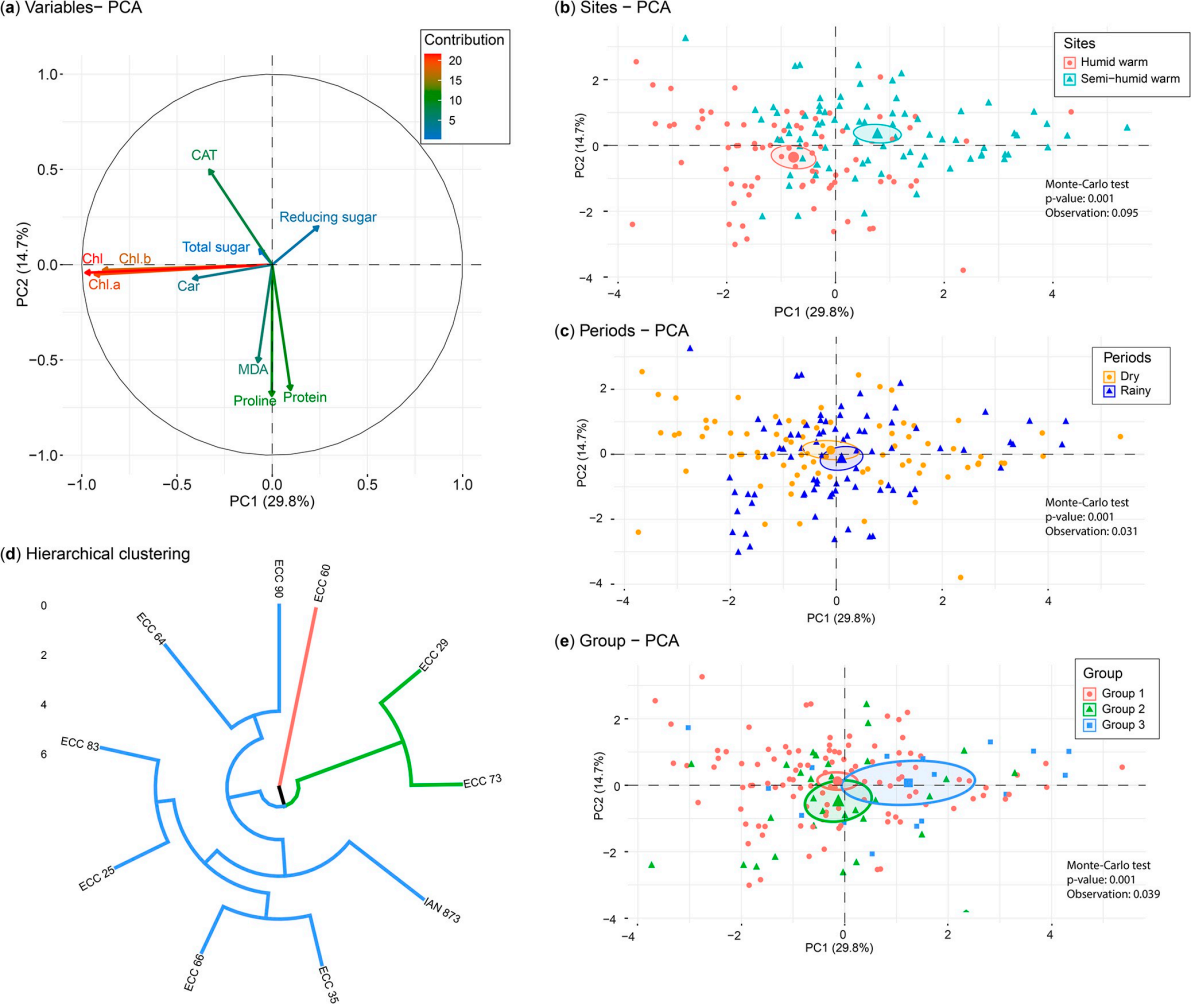
Principal Component Analysis (PCA) of foliar biochemical variables by season and location projected on the PC1/PC2 ordination plan. (a) Correlation circle of the foliar biochemical variables; the color of the vectors indicates the contribution of the variables to the PCs. (b) Location: warm humid (El Paujil) and warm semi-humid (San Vicente del Caguán). (c) Season: dry and rainy. (e) Hierarchical grouping for the classification of the 10 genotypes. (d) Classification groups of the 10 genotypes.

The principal component analysis explained 44.5% of the total variability of the data (F1: 29.8%; F2: 14.7%). According to the Monte Carlo test, a significant effect of biochemical variables was found in relation to location, season, and genotypes (Fig 4A–4C and 4E). The PC1 linked the humid warm site with the content of Chl a, Chl b, total Chl, and carotenoids (Fig 4A and 4B), while PC2 associated the semi-humid warm site with a higher concentration of reducing sugars. At the period level, the highest concentration of total sugars and catalase was attributed to the dry season in PC1, while MDA, proline, and protein were associated with the rainy season in PC2 (Fig 4A and 4C).

Cluster analysis of the variables evaluated in the 10 rubber tree genotypes identified three distinct groups in response to the biochemical characteristics by climatic and seasonal changes (Fig 4D). These groups were generated by their differences (*p* < 0.0001) in the values of chlorophyll content (Chl a, Chl b, and total Chl), carotenoids (Car), proline, catalase (CAT), reducing sugars, total sugars, malondialdehyde (MDA), and protein. The PCA (Fig 4A and 4E) clearly separated groups 1 (ECC 60) and 3 (ECC 90, ECC 64, ECC 83, ECC 25, ECC 66, ECC 35, IAN 873) with PC1 (14.7% of explained variability) with contrasting differences in characteristics related to reducing sugars, total sugars, and carotenoids. The PC2 (29.8% of explained variability) separated group 2 (ECC 29 and ECC 73) with higher concentrations of MDA, proline, protein, and carotenoids.

**Fig 5:**
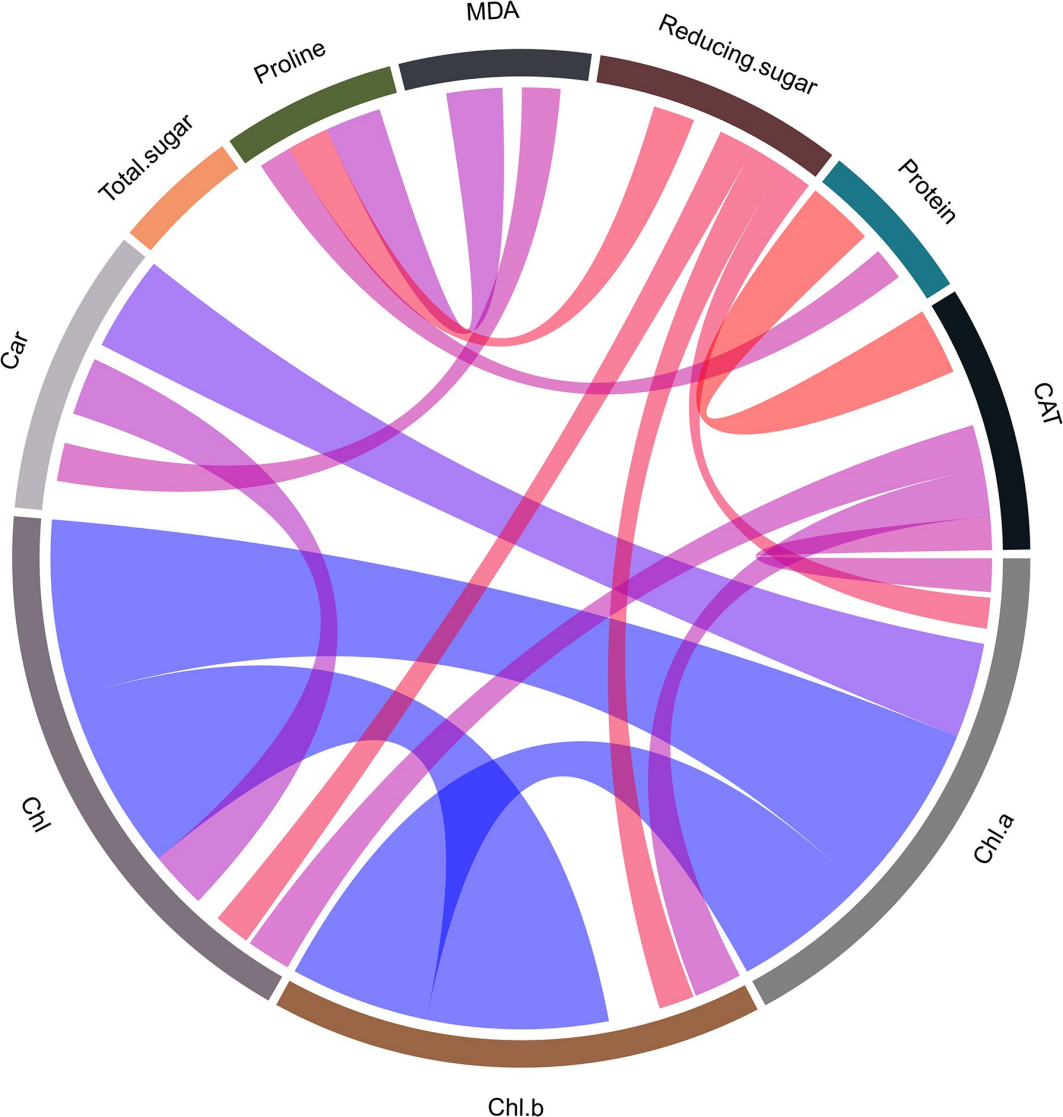
Chord diagram of Pearson correlation coefficients between biochemical variables of 10 genotypes of rubber tree (*Hevea brasiliensis*). Blue bands indicate positive coefficients and red bands indicate negative coefficients. Chlorophyll a, b (Chl a; Chl b) (mg g^-1^), total chlorophyll (Chl) (mg g^-1^), carotenoids (Car) (mg g^-1^), proline (μg g^-1^), catalase (CAT) (Ucat/mg protein), reducing sugars (μg mg^-1^), total sugars (μg g^-1^), protein (mg g^-1^), and malondialdehyde (MDA) (nmol ml^-1^).

The chlorophyll a and b content in rubber leaves showed a highly significant linear correlation (*r* = 0.92, *p*<0.05 and *r* = 0.93, *p*<0.05, respectively) with the total chlorophyll content (Fig 5). Catalase showed a positive correlation with photosynthetic pigments (*r* = 0.17, *p*<0.05; *r* = 0.25, *p*<0.05; and *r* = 0.23, *p*<0.05, respectively) while reducing sugars were negatively correlated with them and with proline content. MDA was positively correlated with proline (*r* = 0.29, *p*<0.05) and carotenoids (*r* = 0.2, *p*<0.05). Furthermore, proline and protein contents were positively correlated (*r* = 0.17, *p*<0.05) (Fig 5).

## Discussion

The results of this study show that the Colombian elite genotypes of *H*. *brasiliensis* have a clear seasonal expression pattern, with higher values of carotenoids, reducing sugars, and malondialdehyde during water deficit conditions (dry season) and higher contents of proline and total sugars during the rainy season. This contrasts with the behavior of a rubber tree under normal conditions, which presents a low level of potentially toxic oxygen metabolites [24] whose production and elimination are controlled through antioxidant defense [27], determining the plant’s plasticity and flexibility in fluctuating conditions [26, 59].

During water deficiency, specifically in the dry period, rubber tree genotypes made adjustments in carotenoid content, which correlate with seasonal CO₂ photosynthetic assimilation [60–63], with greater accumulation reaching its peak during this period [64, 65]. These act as molecular antioxidant protectors [66] and defenders of chlorophyll pigments (Table 2), inhibiting oxidative damage by degrading proteins in the chloroplast membrane through singlet oxygen generation [67], thus protecting the photosynthetic apparatus. Consequently, the possible limited water availability had an effect on the accumulation of reactive oxygen species, causing oxidative stress in leaf cells, lipid peroxidation in membranes, and the development of a better osmotic adjustment capacity by accumulating higher amounts of reducing sugars [5].

The high content of carotenoids and soluble sugars indicated a higher tolerance of genotypes ECC 64 and the control (IAN 873) under water deficit conditions (regardless of location), contributing to the regulation and maintenance of physiological processes within the plant, results that agree with those reported by Chen et al. [68], Wang [12], Gharbi et al. [69], Oliveira dos santos [5] but contrary to Pasaribu et al. [70] indicating high concentrations of total sugars and proline in dry season. Water scarcity does not elicit the same metabolic responses and varies within the same species.

The biochemical response during the rainy period showed the highest contents of proline (ECC 66, ECC 73, and IAN 873) and total sugars (ECC 64, ECC 35, and ECC 25), possibly attributed to nutrient reserves in the form of carbohydrates and energy to be transferred and used for leaf development and shoot growth [32]. However, the higher proline content is due to the level of stress not exceeding the threshold that could trigger the overexpression of genes responsible for its biosynthesis [71]. This is considered a post-stress action [30, 72], presented in the period of lower precipitation, suggesting that environmental factors determine the plant’s capacity to produce this amino acid [73] and its accumulation does not depend solely on abiotic stress.

The proportion of optimal hours of sunlight, favorable temperature, and availability of water in the soil in the rainy season and in the semi-humid climate (San Vicente del Caguán) was found to have the highest chlorophyll a content (Fig 2B). In contrast, during the dry season, the highest concentration of carotenoids was observed (Fig 2D). However, in the more humid climate (El Paujil), the response of the genotypes did not vary with seasonal change except for genotype ECC 60, which was varied at the same site (Fig 2A) during the dry season. Nevertheless, variations in pigment content in the leaves of elite rubber genotypes within the same site varied significantly due to environmental conditions (Fig 2A and 2B) [74] of temperature, humidity [60], water availability [75], and photosynthetically active radiation (Fig 1). Thus, the content and behavior of Chl a significantly differ (*p*<0.05) in genotypes ECC 35, ECC 66, ECC 83, and ECC 90 (Fig 2B) with seasonal change such as rainfall at the same site while genotypes ECC 35, ECC 64, ECC 66, ECC 73, ECC 83, and ECC 90 did not differ seasonally but were different at the same site (Fig 2A). In both sites, the ECC 60 genotype is particularly different for the period with the highest Chl a concentration during the dry period for the two sites, demonstrating that under high irradiance (rain periods for both sites), there is chlorophyll a degradation, suggesting that this pigment seems to be a determining factor for acclimation and a sensitive indicator of photooxidation.

## Conclusions

This study suggest that the rubber trees of the ECC-1 selection of *H*. *brasiliensis* in the growth stage are susceptible to water depletion, as the concentration of biochemical parameters was significantly influenced by seasonal changes in climatic conditions. During the dry period, there is antioxidant protection and defense of the photosynthetic apparatus, while during the rainy period, a nutrient conservation strategy is observed as a post-stress action. In the humid warm site (El Paujil), there was greater ROS production, triggering activation of defense pathways for Chl a, Chl b, and total Chl during the dry period in genotypes ECC 90, ECC 64, and ECC 66, and the highest concentration of proline in genotypes ECC 73, ECC 66, IAN 873, and MDA in genotypes ECC 90, ECC 64, and ECC 60 during the rainy period (higher PAR and precipitation) compared to the semi-humid warm site (San Vicente del Caguán). The ECC 64 genotype, regardless of seasonal change and climatic variation, showed the highest contents in Chl a, total Chl, reducing sugars, total sugars, and MDA, indicating a tendency to adapt to fluctuating conditions. On the other hand, the elite genotypes ECC 90, ECC 64, ECC 35, and ECC 66 stood out for their tolerance to water deficit, showing greater antioxidant responses compared to the IAN 873 (control).

Chlorophyll a and carotenoids play an important role in osmoregulation during water deficit in juvenile rubber trees, proving to be useful assays for identifying tolerance genotypes. This study provides a differential biochemical approach to antioxidant defense capacity under contrasting conditions for juvenile rubber cultivars, contributing to their selection for adaptation and resistance to seasonal changes for local and regional planting and production purposes.
